# Molecular Signatures of Neuroinflammation Induced by αSynuclein Aggregates in Microglial Cells

**DOI:** 10.3389/fimmu.2020.00033

**Published:** 2020-01-31

**Authors:** Souvarish Sarkar, Eric B. Dammer, Emir Malovic, Abby L. Olsen, Syed Ali Raza, Tianwen Gao, Hailian Xiao, Danielle L. Oliver, Duc Duong, Valerie Joers, Nicholas Seyfried, Meixiang Huang, Thomas Kukar, Malú G. Tansey, Anumantha G. Kanthasamy, Srikant Rangaraju

**Affiliations:** ^1^Department of Pathology, Brigham and Women's Hospital and Harvard Medical School, Boston, MA, United States; ^2^Department of Biomedical Sciences, Iowa State University, Ames, IA, United States; ^3^Department of Biochemistry, Emory University, Atlanta, GA, United States; ^4^Department of Neurology, Brigham and Women's Hospital and Harvard Medical School, Boston, MA, United States; ^5^Department of Neurology, Emory University, Atlanta, GA, United States; ^6^Department of Neuroscience, University of Florida College of Medicine, Gainesville, FL, United States; ^7^Department of Pharmacology and Chemical Biology, Emory University, Atlanta, GA, United States

**Keywords:** synuclein, microglia, neuroinflammation, Parkinson's disease, proteomics

## Abstract

Alpha-synuclein (αSyn_Agg_) are pathological hallmarks of Parkinson's disease (PD) and other synucleinopathies that induce microglial activation and immune-mediated neurotoxicity, but the molecular mechanisms of αSyn_Agg_-induced immune activation are poorly defined. We performed quantitative proteomics by mass spectrometry coupled with PCR, immunohistochemical and functional validations studies to define the molecular characteristics of alpha synuclein mediated microglial activation. In mouse microglia, αSyn_Agg_ induced robust pro-inflammatory activation (increased expression of 864 genes including *Irg1, Ifit1*, and *Pyhin*) and increased nuclear proteins involved in RNA synthesis, splicing, and anti-viral defense mechanisms. Conversely, αSyn_Agg_ decreased expression several proteins (including Cdc123, Sod1, and Grn), which were predominantly cytosolic and involved in metabolic, proteasomal and lysosomal mechanisms. Pathway analyses and confirmatory *in vitro* studies suggested that αSyn_Agg_ partly mediates its effects via Stat3 activation. As predicted by our proteomic findings, we verified that αSyn_Agg_ induces mitochondrial dysfunction in microglia. Twenty-six proteins differentially expressed by αSyn_Agg_ were also identified as PD risk genes in genome-wide association studies (upregulated: Brd2, Clk1, Siglec1; down-regulated: Memo1, Arhgap18, Fyn, and Pgrn/*Grn*). We validated progranulin (PGRN) as a lysosomal PD-associated protein that is downregulated by αSyn_Agg_ in microglia *in-vivo* and is expressed by microglia in post-mortem PD brain, congruent with our *in vitro* findings.

**Conclusion:** Together, proteomics approach both reveals novel molecular insights into αSyn-mediated neuroinflammation in PD and other synucleinopathies.

## Introduction

Chronic and sustained microglial activation is a key pathophysiological hallmark of multiple neurodegenerative disorders including Parkinson's disease (PD) (1). Neuroinflammation has been shown to be a key contributor to loss of dopaminergic neurons in animal models of PD (2, 3) and is also observed in striatal and cortical regions of the brain in post-mortem PD studies (4–8). A role for inflammation in PD was first suggested in 1988 when major histocompatibility complex molecules were shown to be upregulated in the brain of PD patients (4). Various pro-inflammatory factors like tumor necrosis factor α (TNF-α) and interleukin 1β (IL-1β) have been shown to be upregulated in cerebrospinal fluid (CSF) and in different regions of the brain in PD patients (9). Moreover, in animal models of PD, such as the 6-hydroxydopamine (6-OHDA), 1-methyl-4-phenyl-1,2,3,6-tetrahydropyridine (MPTP), and rotenone models, selective loss of dopaminergic neurons is accompanied by chronic neuroinflammation (10–13). Human genome wide association studies (GWAS) have identified innate and adaptive immune genes as risk factors for PD (14, 15). Furthermore, treatment with non-steroidal anti-inflammatory drugs (NSAIDs), like ibuprofen, may be effective against PD-related inflammation (16) suggesting that neuroinflammation may modify the course of neurodegeneration in PD.

Microglia, the innate immune responders of the CNS, are key mediators of neuroinflammation in neurodegenerative diseases (2). Depending on the stimuli or disease context, microglia can produce both anti-inflammatory and pro-inflammatory factors, reactive oxygen species (ROS), and growth factors. During development, microglia are also involved in pruning neuronal synapses (17–20), thereby tightly regulating neuronal physiology and survival (21). Pathological αSynuclein (αSyn) aggregation in PD can induce microglial activation and dysfunction. One of the key pathological proteins involved in PD is αSynuclein (αSyn) (22). Misfolded αSyn forms aggregates (αSyn_Agg_), which are the major constituents of Lewy bodies and Lewy neurites, both key neuropathological hallmarks of PD (23). αSyn_Agg_ have been shown to strongly drive the microglial neuroinflammatory response in the diseased brain (22). αSyn_Agg_ has been shown to be phagocytosed by microglia, leading to NADPH oxidase activation and ROS generation, in turn leading to the production of pro-inflammatory, neurotoxic cytokines and chemokines (22, 24, 25). Recent studies have further shown that αSyn_Agg_ can bind to toll like receptor 2 and 4 (TLR2 and TLR4) and cluster of differentiation 36 (CD36) and thereby lead to receptor mediated activation of inflammatory signaling cascades (26, 27). Very recently, we demonstrated the αSyn_Agg_ activates NLRP3 inflammasome through Fyn dependent signaling using cell culture, animal models and human tissues (28). Although αSyn_Agg_ have been shown to induce microglial activation, the integrated molecular pathways and signaling mechanisms involved have not been clearly delineated. A better understanding of the molecular and signaling mechanisms that drive chronic neuroinflammation in PD may provide mechanistic and therapeutically-relevant insights in PD and other αSynucleinopathies.

In this study, we have used quantitative proteomics by mass spectrometry to characterize the proteome-level alterations induced by αSyn_Agg_ in microglia, to identify potential molecular mechanisms of neuroinflammation in PD, including increased Stat3 signaling, increased mitochondrial dysfunction and ribosomal biogeneis and suppression of mitochondrial oxidative phosphorylation. By further comparing our results with existing microglial proteomic datasets, we have identified similarities between LPS- and αSyn_Agg_-induced microglial activation, as well as molecular mechanisms that are unique to αSyn_Agg_-induced microglial activation. To identify αSyn_Agg_-induced microglial protein alterations that are most relevant to human PD, we cross-referenced αSyn_Agg_-regulated proteins in our data with PD risk genes previously identified in human GWAS studies and compared our proteomic results with transcriptomic data from human PD brain. Among the microglial proteins differentially regulated by αSyn_Agg_, we identified novel immune roles for PD-risk genes including progranulin (*Grn*) which appears to be downregulated in microglia in response to αSyn_Agg_. Collectively, these data reveal key molecular signatures of αSyn-induced microglial activation and highlight new disease mechanisms in microglia that may contribute to neurodegeneration in PD and other αSynucleinopathies.

## Materials and Methods

### Cell Culture and Treatments

Primary microglial cells were isolated from postnatal mouse pups (P0-3) following our published protocol (29). Following isolation of microglia from mixed glial culture, cells were treated with 1 μM αSyn_Agg_ for 24 h.

Mouse microglial cell (MMC) line was a kind gift from Dr. Golenbock from University of Massachusetts (30). The MMC line was chosen over other microglial cell lines for proteomic studies due to its similarity with primary microglia as shown previously by our group (31). We have previously shown that MMC at basal level is in a relatively resting/quiescent state which becomes activated by LPS or αSyn_Agg_ treatment to an M1-like state, similar to that seen in primary microglia. Due to the greater resemblance to primary microglia, the MMC line appears to be a better choice than the more commonly used immortalized microglia cell line, BV2 (32). MMC were grown in 10% fetal bovine serum, DMEM, 1% penicillin/streptomycin and 1% glutamate. Treatments were performed in 2% FBS-containing media. Cells were treated with 1 μM αSyn_Agg_ for 24 h (32).

### Recombinant Human αSynuclein Purification and Aggregation

Recombinant αSyn was prepared following a previously published protocol (19, 33). Briefly, transformation with plasmid encoding human αSyn was performed in *E. coli* cells (BL21(DE3) strain) cells. Recombinant αSyn expression was induced by using isopropyl β-D-1-thiogalactopyranoside (IPTG) (Invitrogen). Cells were lysed and recombinant αSyn was purified as previously described (34, 35). We used FPLC system from Biorad to purify the protein and the FPLC chromatogram showed one peak suggesting the purity of the protein (Supplemental Figure 1A). Further, we performed Krypton stain (Supplemental Figure 1B) to determine the purity of the protein. For αSyn aggregation, recombinant protein solution was shaken at a speed of 1000 rpm at 37°C for 7 days (36). The level of endotoxin in αSyn preparations was quantified and <5 EU was detected. Moreover, we confirmed the conformation of the aggregates by electron microscopy (28).

### Animal Studies

All animals were housed under standard conditions of constant temperature (22 ± 1°C), humidity (relative, 30%), and a 12-h light/dark cycle. Use of the animals and protocol procedures were approved by the Institutional Animal Care and Use Committee (IACUC) at Iowa State University (ISU), Ames, IA, USA. αSyn_Agg_ pre-formed fibrils (αSyn_PFF_) were in injected in C57/BL mice bred in our animal facility. Mice were anesthetized as previously described and then injected with 5 of μg αSyn_PFF_ or vehicle. The coordinates indicating distance (mm) from bregma were: AP 0.5, ML 1.9, and DV 4 (28).

### Quantitative Proteomics of Mouse Microglia by Liquid Chromatography Coupled to Tandem Mass Spectrometry (LC-MS/MS)

Samples were prepared essentially as described with slight modifications (37). MMCs were grown to 75% confluence, exposed to αSyn_Agg_ (1 μM) for 24 h, and then harvested. Each cell pellet was individually homogenized in 300 μL of urea lysis buffer (8 M urea, 100 mM NaHPO_4_, pH 8.5), including 3 μL (100 × stock) HALT protease and phosphatase inhibitor cocktail (Pierce) (20, 37). After lysis for 30 min at 4°C, protein supernatants were transferred to 1.5-mL Eppendorf tubes and sonicated (Sonic Dismembrator, Fisher Scientific) three times for 5 s with 15 s intervals of rest at 30% amplitude to disrupt nucleic acids and subsequently vortexed. Protein concentration was determined by the bicinchoninic acid (BCA) method, and samples were frozen in aliquots at −80°C. Protein homogenates (100 μg) were diluted with 50 mM NH_4_HCO_3_ to a final concentration of <2 M urea and then treated with 1 mM dithiothreitol (DTT) at 25°C for 30 min, followed by 5 mM iodoacetimide (IAA) at 25°C for 30 min in the dark. Protein was digested with 1:100 (*w*/*w*) lysyl endopeptidase (Wako) at 25°C for 2 h and further digested overnight with 1:50 (*w*/*w*) trypsin (Promega) at 25°C. Resulting peptides were desalted with a Sep-Pak C18 column (Waters) and dried under vacuum. For LC-MS/MS analysis, derived peptides were re-suspended in 100 μL of loading buffer (0.1% formic acid, 0.03% trifluoroacetic acid, 1% acetonitrile). Peptide mixtures (2 μL) were separated on a self-packed C18 (1.9 μm, Dr. Maisch, Germany) fused silica column (25 cm × 75 μM internal diameter (ID); New Objective, Woburn, MA) by a Dionex Ultimate 3000 RSLCNano and monitored on a Fusion mass spectrometer (Thermo-Fisher Scientific, San Jose, CA). Elution was performed over a 2 h gradient at a rate of 400 nL/min with buffer B ranging from 3 to 80% (buffer A: 0.1% formic acid in water, buffer B: 0.1% formic acid in acetonitrile). The mass spectrometer cycle was programmed to collect at the top speed for 3-s cycles. The MS scans (400–1,600 m/z range; 200,000 AGC; 50 ms maximum ion time) were collected at a resolution of 120,000 at 200 m/z in profile mode, and the HCD MS/MS spectra (0.7 m/z isolation width; 30% collision energy; 10,000 AGC target; 35 ms maximum ion time) were detected in the ion trap. Dynamic exclusion was set to exclude previously sequenced precursor ions for 20 s within a 10 ppm window. Precursor ions with +1 and +8 or higher charge states were excluded from sequencing.

Raw data files were analyzed using MaxQuant v1.6.3.4 with Thermo Foundation for RAW file reading capability, as previously published (20). The search engine Andromeda was used to build and search a concatenated target-decoy IPI/Uniprot mouse reference (downloaded Aug 14, 2015, with human alpha synuclein sequence added, Uniprot ID P37840). Protein methionine oxidation (+15.9949 Da) and protein N-terminal acetylation (+42.0106 Da) were variable modifications (up to five allowed per peptide); cysteine was assigned a fixed carbamidomethyl modification (+57.0215 Da). Only fully tryptic peptides were considered with up to two miscleavages in the database search. A precursor mass tolerance of ±20 ppm was applied prior to mass accuracy calibration and ±4.5 ppm after internal MaxQuant calibration. Other search settings included a maximum peptide mass of 6000 Da, a minimum peptide length of six residues, and 0.05 Da Tolerance for orbitrap (FTMS) HCD MS/MS scans. Co-fragmented peptide search was enabled to deconvolute multiplex spectra. The false discovery rate (FDR) for peptide spectral matches, proteins, and site decoy fraction were all set to 1%. Quantification settings were as follows: re-quantify with a second peak finding attempt after protein identification has completed; match full MS1 peaks between runs; a 0.7-min retention time match window was used after an alignment function was found with a 20 min RT search space. The label-free quantitation (LFQ) algorithm in MaxQuant (21, 22) was used for protein quantitation. Data are available via ProteomeXchange with identifier PXD013691.

### qRT-PCR

RNA isolation from primary microglial cells was performed as described previously (38, 39). Total RNA concentration was measured, and 1 μg RNA was converted to cDNA using the Affinity Script qPCR cDNA synthesis system (Agilent Technologies). Real-time PCR was performed with the RT2 SYBR Green master mix (Thermo-Fisher #K0172). The housekeeping gene 18s rRNA (Qiagen #PPM57735E) was used as the reference for all qRT-PCR experiments. The ΔΔCt method was used, implementing the threshold cycle (Ct) value for the housekeeping gene and for the respective gene of interest in each sample (18, 39). The primers were generated using primer bank (40). The primers were synthesized at Iowa State DNA facility (see Table 1 for primer list).

**Table 1:** List of primers used in experiments.

**Gene**	**Forward primer**	**Reverse primer**
*Fyco1*	CAGTCGAGGACAGCATTGG	GCTCCTTCGCCATGTTCTCA
*Brd2*	AATGGCTTCTGTACCAGCTTTAC	CTGGCTTTTTGGGATTGGACA
*Spast*	CGGCCTGACCGATGTAGAC	TAGCTCCCGTGTCACCTCTTC
*Clk3*	TGTCACAAACGCCGTACCAG	ACGATCTCATATCGCTCTTGGA
*Pmvk*	AAAATCCGGGAAGGACTTCGT	AGAGCACAGATGTTACCTCCA
*Scarb2*	AGAAGGCGGTAGACCAGAC	GTAGGGGGATTTCTCCTTGGA
*Golga3*	AGACCTTCAGTTGTCCCTTGA	GCAGTGGAGCCTGTAGAGG
*Camk2d*	TCCAGAAGTCCTGCGTAAAGA	CCACCAGCAAGATGTAGAGGAT
*Rnps1*	AGAGCTTGCTAGGAGTCAAAGA	TCTCTGCCACGATCCTTCTCA
*Ktn1*	GAGTCCAAAGACCTTCTGAAGAG	TTCTGCAAGGACCGACTTGTA
*Siglec1*	CAGGGCATCCTCGACTGTC	GGAGCATCGTGAAGTTGGTTG
*Fam175b*	CATCTCTACCGCCAACAATTCT	TGGCTAGTATTGCCTAGATTGGG
*Naglu*	ACCGCTATTACCAGAATGTGTG	GTGTGCAAGTTACCCATGCG
*Ubxn4*	GCCATCGCGTCTGCTAAGAG	TGTTTGATGATGCTTGTGTCACT
*Grn*	ATGTGGGTCCTGATGAGCTG	GCTCGTTATTCTAGGCCATGTG
*Tpp2*	TGTCAAAGACTGAGCTTGGAAAG	TGTTGGTGGAGGTATGAGATAGT
*Itpa*	GGAGGAGGTCATTCAGATTCTCG	CTCCCGACACTTCTGTATGGA
*Isyna1*	CGGCCCTCAGTCTACATTCC	ATGTCCTTTCGGATTTGCTCC
*Fam49b*	AAAGTTTTGACATGCACAGACCT	GGATTGCCTCTCGTATTTCGTG
*Psmb9*	CATGAACCGAGATGGCTCTAGT	TCATCGTAGAATTTTGGCAGCTC
*Hspa1*	GCCAAACGGTTCATCGGGA	AGGTGCTATTACCAGCAAGGT
*Hspa1l*	TCACGGTGCCAGCCTATTTC	CGTGGGCTCATTGATTATTCTCA
*Blnk*	GCCCTCCAAGTGTTCCTCG	GGCAGGCATCACATACATCTC
*Arhgap18*	TCGGGAGTTGTGCTAACTGC	GGCCATATCTGCGACTGGAG
*Memo1*	GGATACACATACTGTGGGTCCT	CAGGGGCACATGATGGGAAG

### MAGMA of Human PD GWAS Studies

To determine if any protein products of PD GWAS targets were enriched in a particular module, we used the single nucleotide polymorphism (SNP) summary statistics from http://www.pdgene.org/ (15) to calculate the gene level association value using MAGMA (15). MAGMA calculates the gene level association value by taking the mean of all the transformed (Z statistic) SNP *P*-values associated with a particular gene and uses a known approximation of the distribution to get the gene association value. MAGMA accounts for linkage disequilibrium (LD) using reference data with similar ancestry. These gene lists were further filtered to select for genes that have a MAGMA defined gene association value > 1.3 (-log*P*-value). For each module in the protein network, the mean GWAS significance value (-log P) was calculated as the enrichment score for the module. Random sampling (10,000 times) of the MAGMA gene list was used to assess the significance of the module enrichment score. The enrichment scores were then scaled by subtracting the mean and dividing by the standard deviation of the random samplings. The *P*-value was calculated as the proportion of samplings that have a scaled enrichment score greater than or equal to the module enrichment score. The psychiatric genomics consortium provides links to various data sets.

### Seahorse Mitostress Test

Seahorse metabolic stress test was performed as described previously using a Xfe24 Seahorse (20, 41). Briefly, primary microglial cells (100,000 per well) were plated in PDL-plated Seahorse 24-well plate. Cells were treated with αSyn_Agg_ (1 μM) for 24 h. For MitoStress test, 0.75 μM oligomycin, 0.75 μM FCCP and 0.5 μM rotenone/antimycin were used. Wave 2.6.0 was used to analyze the data.

### Immunocytochemistry

Immunocytochemical analysis was performed per previously published protocols (42). Briefly, primary microglia were isolated and plated on poly-D-lysine coated coverslips and treated. Following treatment, cells were fixed with 4% paraformaldehyde, and blocked with 2% BSA, 0.5% TritonX and 0.05% Tween. The cells were then incubated with primary antibody overnight, washed with PBS, incubated in secondary antibody. The following primary antibodies were used: STAT3 (Cell Signaling Technologies) and pSTAT3 (Y705) (Cell Signaling Technologies).

### Statistical and Bioinformatics Considerations

Differential expression analyses of proteomic data were performed using pairwise *t*-test applied to log2-transformed expression data and adjusted for multiple comparisons using the Benjamini-Hochberg method. For comparisons across more than 2 groups, one-way ANOVA with Tukey post analysis was used. For 2 groups students *t*-test were performed using graphpad prism 5.0. Volcano plots were plotted with the ggplot2 package in R. Proteins with missing data were filtered for minimum criteria as described in results, and missing LFQ abundances were imputed according to an in-house implementation of the Perseus algorithm (43, 44) in R. Gene Ontology (GO) enrichment analyses were performed using GO-Elite software as previously described using input lists of differentially expressed proteins that were either increased or decreased following αSyn_Agg_ exposure. Pathway analyses were also performed (Metacore, Thompson Reuters) as previously described (37).

### Immunohistochemistry Studies of Post-Mortem PD Brain

#### Tissue Preparation

Post-mortem human brain tissue was obtained from the Emory Neuropathological Core (3 cases with PD and 3 non-disease age-matched healthy controls). Immunohistochemical studies were performed on 5-μm paraffin-embedded substantia nigra pars compacta (SNPc) and prefrontal cortex (PFC) sections to detect PGRN and IBA1 (pan-microglial marker) immunoreactivity. Tissue sections were deparaffinized twice by xylene and then hydrated. Deparaffinized sections were then treated with 0.2% Triton-X in a 1X Phosphate Buffered Saline (PBS) solution (pH 7.4) for 1 h at 37°C.

#### Antigen Retrieval

Permeabilized sections were placed in glass slide racks and submerged in a Wheaton dish (Cole Parmer) that contained 250 mL of sodium citrate buffer (85°C, pH 4.5). Wheaton glass dish was then placed into a large Pyrex dish filled with 500 mL of deionized water (85°C) that served as the water bath. Heat mediated antigen retrieval was done by heating slides in a 1450W microwave (Emerson) for 13 min at 60% power.

#### Immunostaining

Hydrogen peroxide (3%) in 60% methanol was used to eliminate endogenous peroxidase activity. These sections were blocked with serum (Jackson ImmunoResearch) in 0.2% Triton-X in 1X PBS for 1 h at 37°C. Sections were then incubated overnight at 4°C with anti-progranulin antibody (R & D Systems; AF2420; 1:400) or anti-IBA1 (Wako; 019-19741; 1:500). Appropriate biotinylated secondary antibodies (Vector Labs) were then applied at 1:500 and incubated for 1 h at 37°C. Staining was performed using the avidin-biotin (Vector Labs) complex method, and tissue developed for 15 min using 3,3′-diaminobenzidine tablets (Sigma-Aldrich).

#### Referenced Microglial Proteomic Datasets

Previously published proteomic data from mouse microglia exposed to LPS were downloaded (37). Differentially expressed proteins in this dataset were compared to αSyn-induced proteomic changes in MMC. We also compared our results in mouse microglia to observations in a previously published transcriptomics study in human PD brain (45).

## Results

### Quantitative Proteomics of Mouse Microglia Reveals αSyn_Agg_-Induced Neuroinflammatory Mechanisms

We first performed qRT-PCR experiments showing that αSyn_Agg_ treatment induced the expression of M1-like pro-inflammatory markers by mouse microglia cell (MMC) line including Nos2, IL-6, TNF, and IL-1β without affecting or decreasing M2 markers such as IRF-4, IGF-1 and MRC1 (Supplemental Figure 2) suggesting that αSyn_Agg_ induces a pro-inflammatory M1-like state *in-vitro*. Moreover, the increased expression of M1-like pro-inflammatory genes were seen only αSyn_Agg_ and not with αSyn monomers. Treatment with monomeric αSyn lead to no significant changes in expression of M1-like markers including Nos2, IL-6, TNF, and IL-1β (Supplemental Figure 3).

To identify proteomic changes in microglia in response to αSyn_Agg_, whole cell lysates of MMCs that had been exposed to αSyn_Agg_ were used for label-free mass spectrometry studies (6 biological replicates per group). MMC lysates in 8M urea buffer were enzymatically digested by trypsin and lysyl endopeptidase C, followed by LC-MS/MS, peptide identification and quantification. In total, we identified 35,725 total peptides (33,957 unique and razor assigned to proteomic database entries) that mapped to 3,816 unique mouse protein IDs and 3,738 unique mouse gene symbols. Of these, 3,345 proteins met inclusion criteria for further analysis (at least 3 non-missing values in either group or at least 2 non-missing values in one group if completely missing in the second group). Missing values in these included proteins were imputed using a R-based script designed to recapitulate the columnwise missing-value imputation algorithm of Perseus (44, 46, 47) commonly applied to MaxQuant LFQ abundances (see Supplemental Table 1).

**Figure 1:**
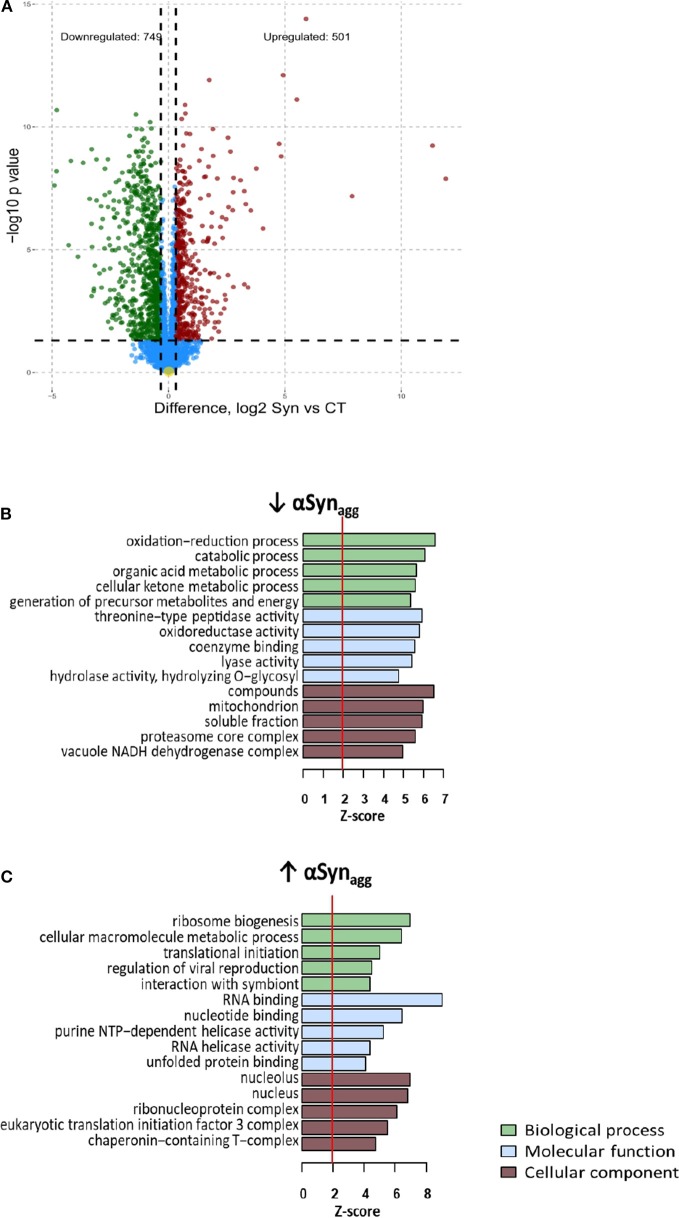
Differential expression analysis of Synuclein-induced proteomic changes in microglia **(A)** Volcano plot: differential expression (Syn vs. Control) **(B)** Enrichment map: Gene ontology analysis of Syn-upregulated proteins **(C)** Enrichment map: Gene ontology analysis of Syn-downregulated proteins For b and c, Node color indicates direction of Syn-induced change in protein expression (Red: Upregulated, Blue: downregulated). Intensity of color of node represents level of significance (darker intensity indicates greater level of significance with white representing 0.05 significance level. Size of node indicates number of genes within the term (range 5–300). Edges connecting nodes are thinnest at a similarity score of 0.4 and max thickness at similarity max 1.

Differential expression analysis comparing αSyn_Agg_-treated with untreated MMCs identified 501 up-regulated and 749 down-regulated proteins (*T*-test unadjusted *p*-value < 0.05 and at least 1.25-fold change in either direction, Figure 1A). Even after adjusting for multi-pairwise comparisons (BH FDR <5%), 1,578 proteins were differentially expressed of which 109 proteins demonstrated at least 4-fold change in either direction (38 up-regulated and 71 down-regulated, see Supplemental Table 1). The top αSyn_Agg_ up-regulated proteins included several pro-inflammatory proteins such as Irg1, Ifit1, and Pyhin1 while αSyn_Agg_-downregulated proteins included Sod1, Ahnak2, Cd93, and Thumpd1.

GO enrichment analyses of αSyn_Agg_-upregulated and downregulated proteins were then performed. Nuclear and nucleolar proteins involved in RNA binding and ribosomal biogenesis, RNA splicing and anti-viral defense responses were highly represented within the αSyn_Agg_-upregulated proteins (Figure 1B). KEGG pathways highly represented within these proteins included ribosome biogenesis, spliceosome, and fatty acid biosynthesis. Conversely, cytosolic proteins involved in oxidation-reduction and catabolic processes, proteasome core complex function and calcium binding were highly enriched within αSyn_Agg_-downregulated proteins (Figure 1B). KEGG pathways enriched in this list included several small molecule metabolic pathways, as well as proteasome and lysosomal pathways. These results suggest that αSyn_Agg_ strongly induces RNA synthesis and splicing while suppressing homeostatic metabolic, mitochondrial, proteasomal, and lysosomal activities.

**Figure 2:**
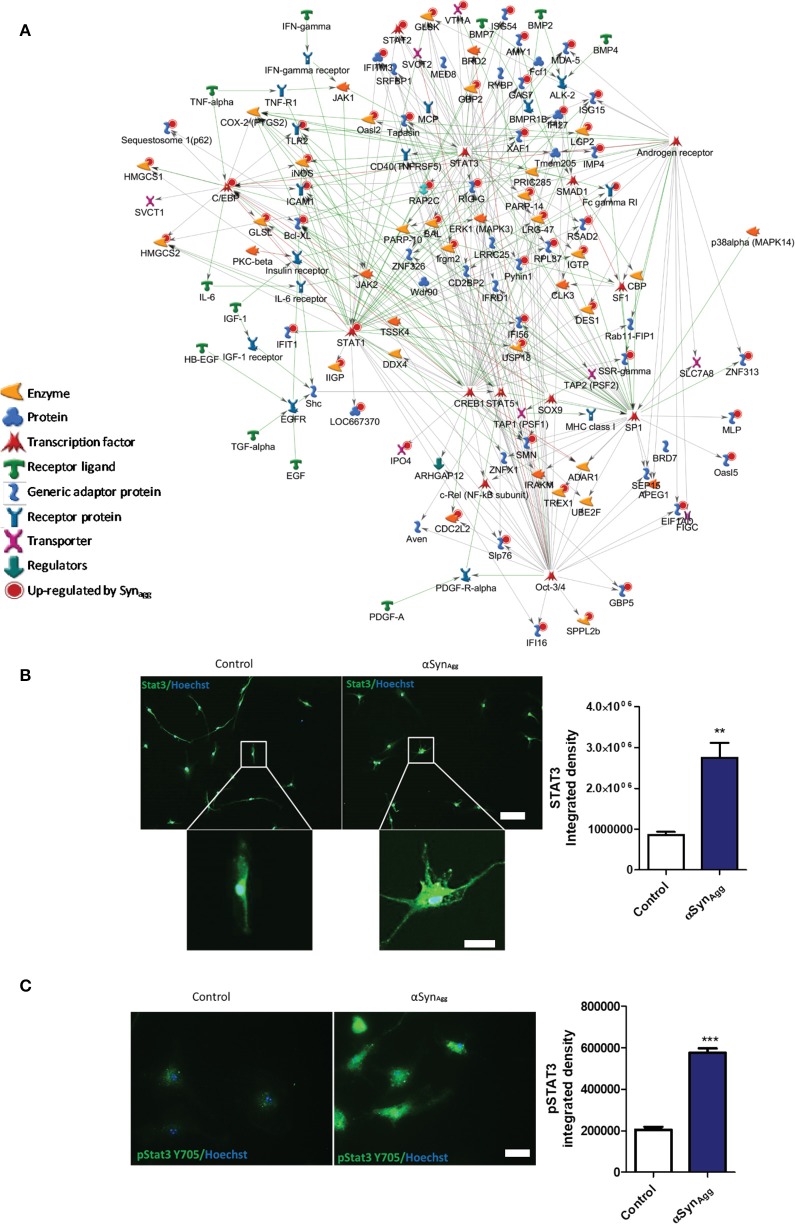
Syn upregulated proteins are downstream of Stat3 and Stat1 signaling pathways. **(A)** Pathway analysis of the top three Syn-upregulated proteins in microglia. Key immune transcriptional factors including Stat3 and Stat1 are highlighted with Red circles. **(B)** Confirmation of Stat3 (Scale bar = 100 μM inset scale bar = 15 μM) and **(C)** pSTAT3 upregulation by Syn in primary mouse microglia. Scale bar = 50 μM. Data analyzed using student's *t*-test with *n* = 4 for each group. **p < 0.01, *** < 0.005.

Canonical pathway analysis (Metacore) revealed that signaling via Stat3, Stat1, Oct3/4, and C/ebp transcriptional pathways are likely to be involved in αSyn_Agg_-mediated regulation of protein expression (Figure 2A). In confirmatory studies in primary microglia, we further observed that αSyn_Agg_ robustly increased both native Stat3 (Figure 2B) and Stat3 tyrosine phosphorylation (Y705) (Figure 2C). Further experiments with monomeric αSyn showed no increase in Stat3 (Supplemental Figure 4A) or pStat3 (Supplemental Figure 4B) protein expression indicating αSyn_Agg_-specificity of microglial responses. Together, we have identified probable pathways that regulate αSyn_Agg_-induced microglial activation and pro-inflammatory mechanisms.

### Identification of Proteomic Changes Unique to αSyn_Agg_-Activated Microglia

**Figure 3:**
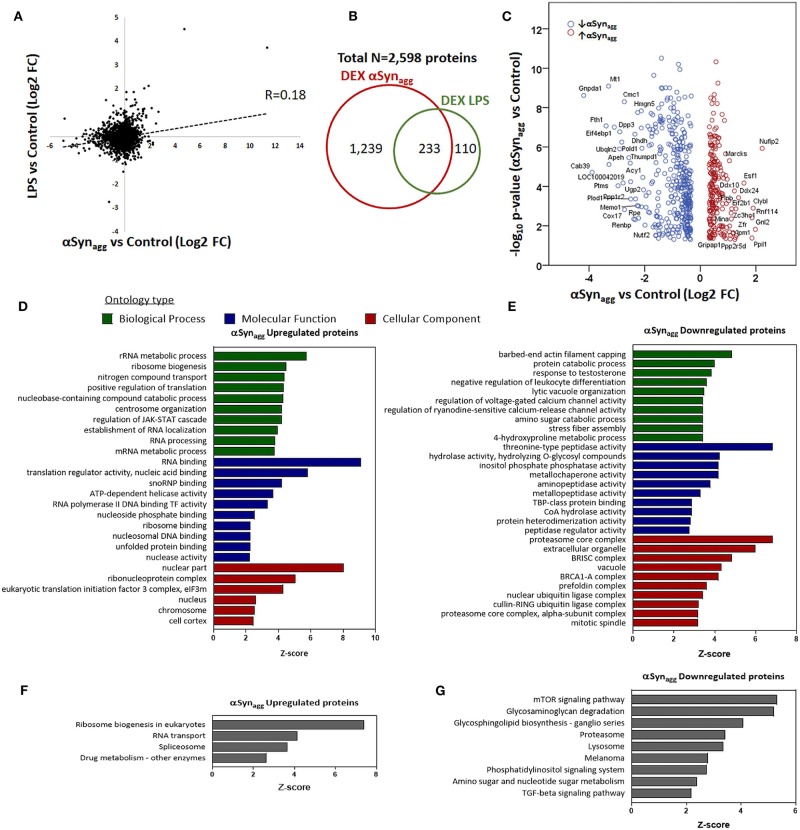
Comparison of Syn-induced and LPS—induced microglial proteomic changes in mouse microglia. **(A)** Correlation between fold-changes in protein expression comparing αSyn_Agg_ vs. control with LPS vs. control microglial proteomic datasets. Pearson's Rho is shown. **(B)** Venn diagram showing degree of overlap between proteins differentially expressed (DEX) in response to αSyn_Agg_ and LPS in mouse microglia. **(C)** Volcano plot of proteins that are differentially expressed in response to αSyn_Agg_ but not by LPS (defined as at least 1.25-fold change and p <0.05 in response to αSyn_Agg_ but *p* > 0.2 in response to LPS). The labeled proteins in the volcano plot are the significant hits. **(D,E)** Gene Ontology enrichment analyses of uniquely Syn-upregulated proteins in microglia (**D**: GO terms, **E**: KEGG pathways). **(F,G)** Gene Ontology analysis of uniquely Syn-downregulated proteins (**F**: GO terms, **G**: KEGG pathways). For panels d-g, only top 10 enriched GO or KEGG pathway terms that met significance criteria (enrichment Z-score>1.96) are shown.

αSyn_Agg_, like LPS, may induce microglial pro-inflammatory activation via TLR signaling (27, 48, 49) but in addition, may have unique effects on microglial activation via distinct mechanisms that are not completely understood. To identify αSyn_Agg_-induced microglial protein changes that overlap with, or are distinct from LPS pro-inflammatory activation of microglia, we compared αSyn_Agg_-induced differentially expressed proteins in this dataset with existing proteomic data from LPS-treated BV2 mouse microglia (37). 2,598 proteins quantified in our dataset were also quantified in this reference mouse microglial proteome comparing LPS-treated to untreated BV2 microglia (Supplemental Table 2) (37). Among these shared proteins, 1,472 were differentially expressed by αSyn_Agg_ (*p* < 0.05) of which 233 proteins were differentially expressed in both LPS and αSyn_Agg_ datasets (unadjusted *p* < 0.05), and overall level of concordance was low (Pearson's *R* = 0.18) (Figure 3A). While majority of LPS-differentially expressed proteins (67.9%) were also differentially expressed following αSyn_Agg_, only 15.8% of αSyn_Agg_-differentially expressed proteins were differentially expressed following LPS stimulation (Figure 3B). Among the shared proteins, the top concordant proteins included Irg1, Saa3, Sqstm1, Ehd1, Nadk, Icam1, and Marcksl1. These results indicate that while αSyn_Agg_ induces an LPS-like pro-inflammatory activation profile in microglia, the majority of αSyn_Agg_-induced changes are distinct from LPS-induced changes.

To define the unique molecular mechanisms regulated by αSyn_Agg_ that are distinct from LPS-induced changes, we performed an analysis restricted to 596 proteins that were only differentially regulated by αSyn_Agg_ but not by LPS (proteins with ≥1.25-fold differential expression [*p* < 0.05] in response to αSyn_Agg_, but *p* > 0.2 for LPS vs. control comparisons) (Figure 3C). GO analysis of 216 αSyn_Agg_-upregulated (but not by LPS) proteins revealed enrichment of nuclear and nucleolar proteins involved in RNA metabolic processes, ribonuclear biogenesis, and splicing (Figures 3D,E). On the other hand, 380 αSyn_Agg_-specific and downregulated proteins (Figures 3F,G) were enriched for cytosolic, extracellular and exosomal proteins involved in proteasomal function, small molecular metabolism, peptidase activity, cellular catabolic processes, mTOR signaling and proteolysis. Overall, these comparative analyses show that while some microglial responses to αSyn_Agg_ are similar to pro-inflammatory effects of LPS, αSyn_Agg_ also uniquely increases the expression of ribonucleoprotein and the RNA binding machinery while suppressing catabolic and protein degradation/proteasomal processes in microglia.

### Identification of αSyn_Agg_-Regulated Proteins in Microglia That Have Pathophysiological Relevance to Human PD

**Figure 4:**
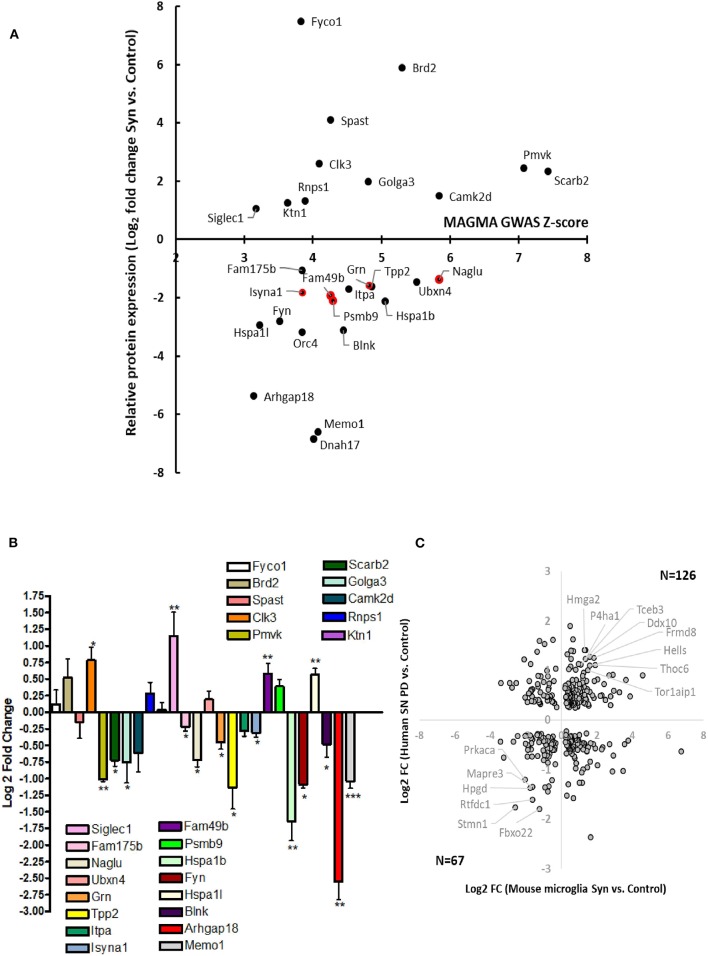
Identification of Syn-induced microglial proteomic changes that are relevant to human PD pathogenesis**. (A)** MAGMA analysis of PD GWAS risk genes that are also differentially expressed in response to Syn. The 5 gene symbols highlighted in red represent proteins that are also most highly expressed in microglia as determined by CNS cell-type-specific proteomics. **(B)** qRT-PCR validation studies of human PD GWAS and Syn-regulated proteins in primary mouse microglia. **(C)** Comparison of results from MMC Syn vs. control proteomics dataset and PD vs. control substantia nigra microarray study. 39 gene symbols showed differential expression at the unadjusted *p* < 0.05 level. Of these 193 (57%) showed concordant changes in both datasets. Gene symbols meeting at 2-fold change in both datasets are highlighted (8 concordantly upregulated and 6 concordantly downregulated).

To derive a comprehensive list of known human PD risk genes identified by GWAS, we performed a meta-analysis of existing GWAS studies using MAGMA, and identified 622 genetic risk factors for PD (Supplemental Table 3) (15). We cross-referenced this list of PD risk genes with our microglia proteomic dataset and identified 28 proteins that were differentially expressed in microglia (≥2-fold change in either direction) in response to αSyn_Agg_ that also met GWAS-level statistical significance (MAGMA *p*-value <0.05) (Figure 4A, Table 2). We then performed qRT-PCR studies (Table 3, Figure 4B) in primary murine microglia after exposure to αSyn_Agg_ using identical experimental conditions to determine whether findings observed in the MMC microglial cell line can be replicated in primary mouse microglia. Of the 26 transcripts evaluated, congruent changes were observed for 3 Syn-upregulated (Brd2, Clk3, Siglec1) and 11 Syn-downregulated (Memo1, Arhgap18, Blnk, Fyn, Hspa1b, Isyna1, Itpa, Tpp2, Grn, Naglu, and Fam175b) proteins (Figure 4B, Table 3).

**Table 2:** PD risk genes demonstrating differential protein expression in microglia following αSyn_Agg_.

**Protein**	**Probable role**
FYCOl	Regulates Phagosome maturation
BRD2	Inhibition of BRD2-4 (BET proteins) leads to decreased inflammation through SIRT activation
SPAST	SPAST was significantly increased in prostate tissue with high inflammation
CLK3	Splicing; CLK1 have been shown to be a target in AD
PMVK	HFD promotes PMVK in CD44 positive cells
SCARB2	Involved in innate immunity and autophagosome maturation
GOLGA3	GOLGA3 decreases ubiquitination of serine racemase. D-serine increases oxidative stress and causes inflammation
CAMK2D	Downstream of Nfkb
RNPS1	Involved in Nonsense mediated deacy
KTN1	Intracellular organelle transport
SIGLEC1/CD169	Neuron glia interaction, plays role in phagocytosis; marker of pathogenic phagocytosis in MS
FAM175B	Regulates IFN pathway
NAGLU	Required for lysosomal degradtion of heparan suphate, leads to neurodegneration
UBXN4	Invovled in ER protein degradation
GRN	Defeciency activates complement pathways and synaptic prunning
ITPP2	Component of the proteolytic cascade acting downstream of the 26 S proteasome in the ubiquitin-proteasome pathway. Release of anN-terminal tripeptide from a polypeptide.
ITPA	Deactivation leads to accumulation of ionosin triphosphate. Mutated in IBD patients
ISYNA1	Modified by nitric oxide post LPS treatment
FAM49B	Silencing leads to mitochondrial fission and ROS generation
PSMB9	Major component of immunoproteosome
HSPA1B	Chaperon involved in stress, KO mouse more vulnerable to PD toxicant MPTP. Deletion causes MAPK activation in cardiomyocytes
FYN	Our group have shown that Fyn phosphorylation leads to inflammation in PD models
HSPA1L	Involved in mitochondrial protein transport and folding
BLNK	Regulates RET signaling
ORC4	Not found
RHGAP18	Downregulation caues formation of stress fibers, downregulated in LPS treated microglia
MEMO1	Involved in migration and microtubule assembly
DNAH17	Microtubule motor activity

**Table 3:** Concordance between differentially expressed genes in human PD and proteins in αSyn_Agg_-treated microglia (related to Figure 4C).

**Protein**	**Role**
HIMGA2	Driver of inflammation in liver toxicity induced by LPS
P4HA1	Knockdown reduces prolifereration and migration of glioma stem cells
FRMD8	Promotes inflammation and growth
STMN1	Regulates transcription
FBX022	Reduces inflammation by inhibition of Nfkb
STMN1	Induces proliferation and is activated by phosphorylation
HPGD	Inhibits prostagladins and hence regulates inflammation
MAPRE3	Regulates microtubule assembly and cellular polarization.

While most risk genes for PD regulate non-immune functions, genes/proteins that are most highly expressed in microglia are also most likely to regulate microglial functions and neuroinflammation in PD. Of 622 PD GWAS risk genes identified by MAGMA, 26 genes were most highly expressed in microglia based on a CNS cell-type-specific proteome from purified mouse microglia, astrocytes, oligodendrocytes and neurons (50). Of these microglial PD-risk genes, 5 proteins (Psmb9, Fam49b, Isyna1, Grn, and Naglu) were also differentially regulated by αSyn_Agg_ in our dataset (Figure 4A). Interestingly, these 5 proteins were all suppressed by αSyn_Agg_ by at least 2-fold, suggesting that polymorphisms in these 5 genes may partly replicate downstream immune effects of αSyn_Agg_. These αSyn_Agg_-regulated and human PD-risk proteins may represent immune genes with causative roles in PD.

We also compared αSyn_Agg_-induced proteomic changes in microglia with an existing gene microarray dataset obtained from the post-mortem samples from the substantia nigra regions of PD and non-PD control patients in which 5,933 genes were differentially expressed (45) of which, 782 gene symbols were also identified in our microglial proteomic dataset. 339 genes/proteins of these 782 demonstrated differential expression in response to αSyn_Agg_ (Figure 4C) although poor concordance between direction of change was observed (rho = 0.1).

### Progranulin (PGRN) Protein Is Expressed by Microglia in Human PD

**Figure 5:**
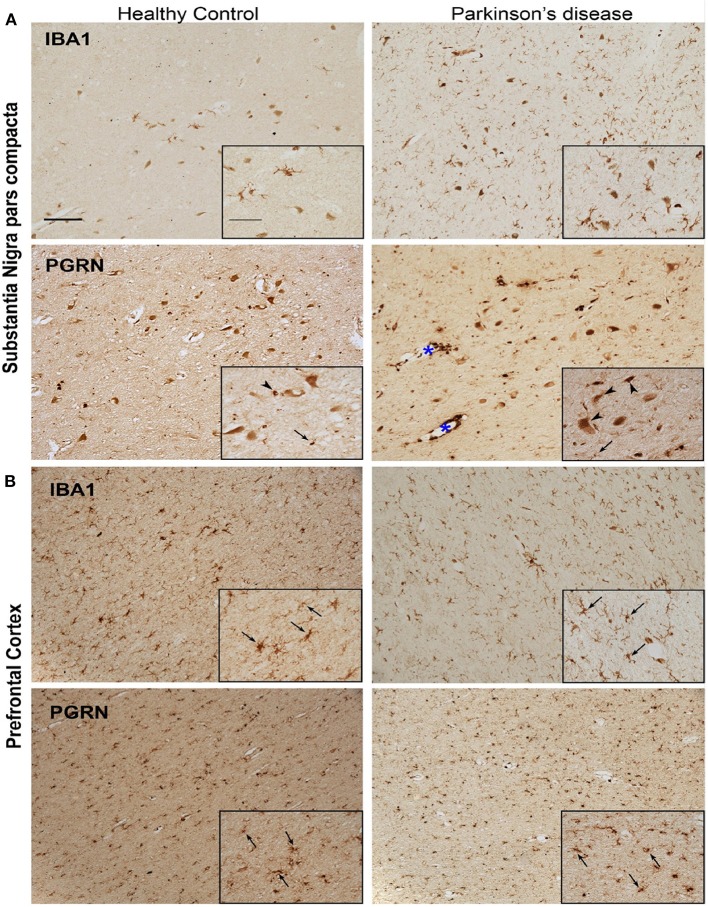
Representative microphotographs of IBA1 and PGRN immunostained tissue in the region of the SNpc **(A)** and PFC **(B)** of a healthy control and PD patient. Presence of IBA1+ microglia significantly increased in the nigra of PD compared to healthy control, yet despite the abundant quantity of microglia, few PGRN-immunoreactive cells resemble microglia morphology (arrows) in the SNpc **(A)**. Instead, PGRN is detected in neuromelanin cells or present as neuronal cell bodies (arrow heads) and in perivasculature spaces (blue asterisk). IBA1-immunoreactivity is similar in the PFC between healthy control and PD patient, and PGRN+ cells largely resemble microglial morphology (arrows) in both subjects **(B)**. Scale bar 100 μm, inset 50 μm.

We observed that PGRN protein levels as well as *Grn* mRNA transcripts were concordantly decreased in mouse primary microglia in response to αSyn_Agg_. Furthermore, Grn is highly expressed at the transcript and protein levels in mouse and human microglia (50, 51) in addition to being identified as a risk gene for PD (15). Therefore, we performed validation studies using an *in-vivo* model of αSyn-aggregate induced neuroinflammation in mice (36, 52). We analyzed brain tissues from mice that received stereotaxic injections with αSyn pre-formed fibrils (αSyn_PFF)_ (Supplemental Figure 5A). In this model, open-field versamax test revealed that αSyn_PFF_ induced motor behavioral changes in mice (Supplementary Figures 5B,C). Further, qPCR analysis of substantia nigra from the injected side validated Grn and other genes which were differentially regulated in our microglial proteomic dataset (Clk3, Golga3, Memo1, and Isyna1) (Supplemental Figure 5D). While these effects observed do not reflect microglia-specific alterations induced by αSyn, future studies will clarify the cell types responsible for these gene expression changes. Next, we determined patterns of PGRN protein expression in post-mortem brain tissues obtained from subjects with PD and age-matched non-disease controls, in SNpc and PFC regions (Figure 5). As expected, we observed the presence of Iba1+ microglia cells with ramified morphology in PFC in both PD and HC brains; whereas the PD SNpc displayed increased numbers of Iba1+ cells as compared to HC SNpc. PGRN immunoreactivity was predominantly observed in the melanized dopaminergic neurons in the SNpc, and to a lesser extent in cells with glial morphology in the SNpc. However, relative to HC SNpc, PD SNpc displayed more PGRN+ inclusions and intense labeling in cells lining vessels which did not have glial morphology. Unlike the SNpc, PGRN immunoreactivity in the PFC was seen in glial cells that predominantly had microglial morphology regardless of disease status. Double immunofluorescence studies of PGRN and microglial markers were confounded by lipofuscin-associated auto-fluorescence, limiting our ability to perform quantitative and microglia-specific analyses. Overall, since Grn is highly expressed at the transcriptomic level in microglia in mammalian brain, our *in-vivo* studies show a general agreement that PGRN expression is indeed observed in microglia in human PD cases, especially in the pre-frontal cortex.

### αSyn Induced Mitochondrial Dysfunction in Microglial Cells

**Figure 6:**
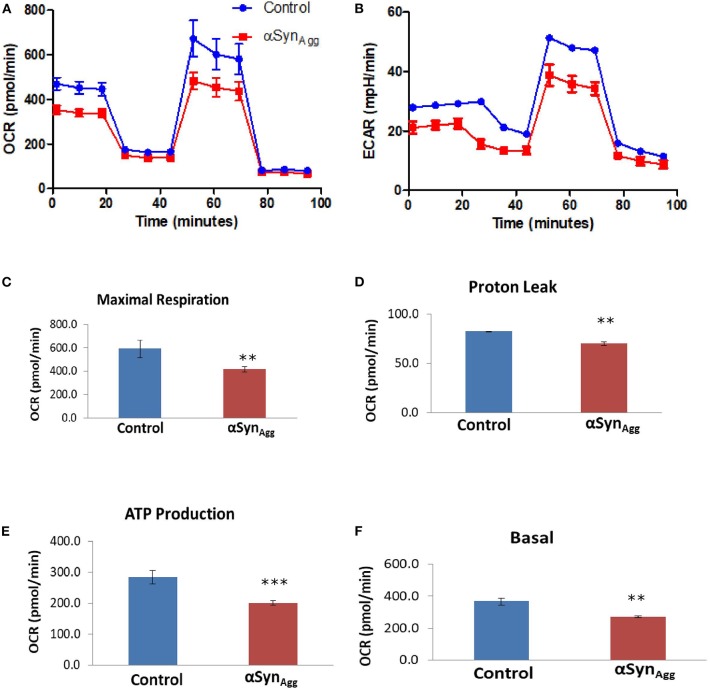
αSyn induced mitochondrial dysfunction in microglial cells. Primary microglial cells were treated with 1 μM αSyn for 24 h and Seahorse Mitostress test was performed. **(A)** OCR and **(B)** ECR of primary microglial cells treated with αSyn. **(C)** Maximal respiration, **(D)** Proton leak, **(E)** ATP production, and **(F)** Basal respiration of primary microglial cells treated with αSyn. Data analyzed using student's *t*-test with *n* = 4 for each group. **p < 0.01, *** < 0.005.

Since our *in vitro* mouse microglia showed concordant dysregulation of mitochondrial proteins induced by αSyn_Agg_, we performed Seahorse studies of mitochondrial stress in primary mouse microglia (Figures 6A,B). Though the role of mitochondrial dysfunction is well studied in neurons (53), the exact function of microglial mitochondrial dysfunction is still not well-understood. We have recently shown that mitochondrial dysfunction in microglia leads to inflammation by activation of NLRP3 inflammasome activation (31). Hence, to further validate that αSyn can induce mitochondrial damage in microglial cells, we performed seahorse mitostress test on primary mouse microglial cells, treated with 1 μM αSyn_Agg_ for 24 h to mirror our proteomic studies. αSyn altered mitochondrial dynamics in microglial cells as shown by changes in maximal respiration (Figure 6C), proton leak (Figure 6D), ATP production (Figure 6E) and basal respiration (Figure 6F). These confirmatory findings, together with our proteomics results, show that αSyn can cause mitochondrial dysfunction in microglial cells.

## Discussion

Neurodegenerative disorders including Alzheimer's disease and PD are characterized by chronic inflammation in the brain (2, 54, 55). Hyperactivation of microglia and astroglia is a key pathophysiological hallmark of these neurodegenerative diseases (55, 56). GWAS have identified multiple immune related candidate genes which can presumably modify PD disease risk (57). αSyn_Agg_ is one of the major components of Lewy body and Lewy neurites. Various studies from our group and others have shown that αSyn_Agg_ activates microglial cells to induce the production of pro-inflammatory cytokines and chemokines (25, 32, 58). αSyn_Agg_ can bind to TLR-2 or CD-36 on microglial surface to activate the downstream pro-inflammatory signaling cascade (59).

Though neuroinflammation is a key pathological finding in PD, the mechanisms involved in αSyn-induced neuroinflammation are not well-defined, in part due to lack of relevant *in vivo* model systems. In this study we have performed comprehensive analyses of microglial protein changes following αSyn_Agg_ treatment to define the key molecular changes and pathways that are activated or suppressed by αSyn_Agg_. Through GO analyses of differentially expressed proteins and by integrating our findings with existing microglial proteomes, we have identified several molecular pathways including Stat3 activation, decreased antigen presentation, increased RNA splicing and mitochondrial oxidative stress as predominant and unique effects of αSyn in microglia. The human disease relevance of our findings is further emphasized by identification of several PD GWAS risk genes within αSyn-regulated proteins. Specifically, we found that progranulin was downregulated both at the mRNA and protein level in mouse microglia in response to αSyn. Consistent with this finding, we observed that relative to the significant upregulation of IBA1+ cells in the SNpc of PD brains, where αSyn aggregates are common, the disease state does not result in greater levels of PGRN+ cells. No marked difference was found between PD and HC PFC in microglia PGRN expression, a brain region where αSyn aggregation is less common in PD. In addition, the reductions in Grn mRNA and PGRN protein in PD may be further compounded by sequestration of PGRN into aggregates or inclusions evident in the PD SNpc in a manner analogous to what has been reported for other endo-lysosomal membrane proteins (LAMP1 and Rab5) accumulating at amyloid plaques (60).

The JAK stat pathway has been implicated in regulating inflammation and neurodegeneration in an αSynuclein adeno-associated virus model of PD pathogenesis (61). This study demonstrated that inhibiting JAK/STAT pathway using an inhibitor reduced MHC-II and inflammatory gene expression in microglia induced by αSynuclein. Further, Qin et al. demonstrated that JAK/STAT inhibitor reduced inflammatory chemokines and cytokines, infiltrating T-cells and microglial activation in rodent models of PD. Here, we demonstrate that αSyn_Agg_ induced the upregulation of pro-inflammatory proteins and among these, the STAT3 signaling pathway was highly enriched in our proteomic analysis. We further validated our findings from proteomics using primary microglial cells and showed that αSyn_Agg_-induced upregulation of both total and phosphorylated STAT3. Interestingly, the effects on STAT3 were specific to the aggregate rather than monomeric form of αSyn emphasizing the profound pro-inflammatory effect of aggregated αSyn on microglia (Supplementary Figure 4). These findings also suggest that the STAT3 signaling pathway may be targeted to reduce αSyn_Agg_-induced inflammation in glial cells.

αSyn_Agg_ can be taken up by microglial cells. Once internalized, αSyn_Agg_ has been shown to bind to mitochondrial pore complex to block the TOM20 pore complex leading to mitochondrial dysfunction and apoptosis in neurons (62). Though the role of mitochondrial dysfunction in neurons is well-established in PD, the role of glial mitochondrial dysfunction is not well-studied. We have recently shown that mitochondrial dysfunction in glial cells can lead to an inflammatory response (19, 20). We also identified metabolic pathways that are altered post αSyn_Agg_ treatment further suggesting the probable role of mitochondria in regulating inflammation. Furthermore, we validated using seahorse metabolic flux assay that αSyn_Agg_ leads to mitochondrial dysfunction.

Another specific signature of αSyn_Agg_-induced changes in microglial cells is modulation in expression of RNA binding proteins. Our GO analysis revealed an enrichment of RNA binding proteins induced by αSyn_Agg_ and not LPS. Though αSyn is classically considered to be a synaptic protein, recent studies have shown that αSyn can localize to the nucleus and regulate histone modification and neurotoxicity (63, 64). Out of the RNA binding proteins which are upregulated, of interest is Caspase8. We have recently shown that αSyn_Agg_ leads to NLRP3 inflammasome activation through Fyn signaling (28) in microglial cells leading to IL-1β release although inhibition of NLRP3 inflammasome did not completely attenuate IL-1β release, presumably indicating NLRP3 inflammasome-independent release of IL-1β. Caspase8 has been shown to regulate IL-1β secretion independent of inflammasome activation depending on the stimulus (65, 66). Further studies focused αSyn_Agg_-induced IL-1β release and Caspase-8 dependent cleavage of IL-1β may identify a novel mechanism that drives microglia-mediated neuroinflammation. Further studies are required to understand the interaction of these RNA-binding proteins and αSyn_Agg_ as well as the exact mechanism that regulates this interaction.

Though GWAS have been able to identify genetic risk factors of PD development, most of the studies relating GWAS hits to their functions have been limited to neurons. In this study we compare GWAS results with microglial proteomics, raising the possibility that some of the risk of PD may be mediated through microglia. The comparison between GWAS studies and our proteomic study identified progranulin as a potential candidate protein that is regulated by αSyn_Agg_. We have recently shown that Fyn, a hit in our proteomic data, is modified in PD patients and plays a role in inflammasome activation (28). Downregulation of progranulin in microglial cells has been shown to induce synaptic pruning through the complement pathway (67). Progranulin has further been shown to be a chemoattractant for microglia and to regulate the endosomal pathways of microglia, which were also altered in our proteomic study (68). We further validated this result in primary microglial cells using qPCR. Furthermore, immunohistochemistry in post mortem brains from PD patients and age matched controls showed PGRN expression in both neurons and microglia; but reduced PGRN immunoreactivity in cells of glial morphology in PD SNpc compared to PD PFC. These results suggest downregulation of PGRN in brain regions with αSyn aggregates/inclusions (60). Interestingly, a recent transcriptomic analysis using PGRN knockout mice have shown that granulin knockdown enhances the microglial neurodegenerative phenotype (MGnD) (69). Further mechanistic studies in animal models and cell culture model of PD to identify the role of PGRN in regulating microglial inflammation in PD is warranted.

In conclusion, we have employed a comprehensive proteomics approach integrated with experimental validation to identify novel molecular mechanisms of αSyn_Agg_-induced neuroinflammation. In mouse microglia, αSynuclein uniquely increases expression of RNA binding proteins suggesting augmented RNA processing and splicing in addition to mitochondrial oxidative stress. We also provide evidence for decreased microglial progranulin as a novel disease mechanism in PD, implicating lysosomal dysfunction and autophagy in PD pathogenesis. A limitation of our study is the lack of mechanistic understanding of neuroinflammation induced by α-synuclein. Further, different synucleinopathies have different forms of aggregates, which may have different levels of toxicity. Future studies looking into the signatures of different form of aggregates is warranted. Our comprehensive quantitative proteomic dataset represents a valuable resource that can guide future neuroscience research to better understand αSyn-mediated neurodegenerative diseases. Future studies demonstrating the mechanistic relationship between some of the proteins identified in this study is necessary to understand the etiology of microglial activation in PD.

## Data Availability Statement

The raw data supporting the conclusions of this study will be made available by the authors upon reasonable request. Data are available via ProteomeXchange with identifier PXD013691.

## Ethics Statement

The animal study was reviewed and approved by Emory IACUC.

## Author Contributions

SS, ED, AK, and SR conceptualized the study, SS, ED, AO, SAR, TG, DO, VJ, HX, EM, NS, and MH performed the experiments. SS, ED, SR, MT, NS, TK, and AK analyzed data and edited the manuscript.

### Conflict of Interest

Patent pending related to this work entitled “Methods to treat neurodegeneration with granulins” to TK. AK is a shareholder of PK Biosciences Corporation (Ames, IA), which is interested in identifying novel biomarkers and potential therapeutic targets for PD. The remaining authors declare that the research was conducted in the absence of any commercial or financial relationships that could be construed as a potential conflict of interest.
