# Supplementary material for: Molecular Signatures of Neuroinflammation Induced by αSynuclein Aggregates in Microglial Cells
Source: Front Immunol. 2020 Jan 31;11:33. doi: 10.3389/fimmu.2020.00033 (PMC7006296; doi:10.3389/fimmu.2020.00033)
Supplement: Supplementary file 4 [file Data_Sheet_4.docx]

**Supplemental Figures**

**Molecular signatures of neuroinflammation induced by α-synuclein aggregates in microglial cells**

**Souvarish Sarkar^1,2^, Eric B. Dammer^6^, Emir Malovic^2^, Abby L. Olsen^7^, Syed Ali Raza^3^, Tianwen Gao^3^, Hailian Xiao^3^, Danielle L. Oliver^5^, Duc Duong^6^, Valerie Joers^5^, Nicholas Seyfried^3,6^, Meixiang Huang^4^, Thomas Kukar^3,4^, Malú G. Tansey^5^, Anumantha G. Kanthasamy^2^,* Srikant Rangaraju ^3^***

1- Department of Pathology, Brigham and Women’s Hospital, Harvard Medical School, Boston, MA

2- Department of Biomedical Sciences, Iowa State University, Ames, IA

3- Department of Neurology, Emory University, Atlanta, GA

4- Department of Pharmacology and Chemical Biology, Emory University, Atlanta GA

5- Department of Neuroscience, University of Florida College of Medicine, Gainesville, FL

6- Department of Biochemistry, Emory University, Atlanta GA

7- Department of Neurology, Brigham and Women’s Hospital, Harvard Medical School, Boston, MA

**Co-corresponding authors: Dr Rangaraju and Dr Kanthasamy contributed equally as c-senior and co-corresponding authors*

Srikant Rangaraju, Department of Neurology, Emory University, Atlanta, GA 30322, USA; [Srikant.rangaraju@emory.edu](mailto:Srikant.rangaraju@emory.edu)

Figures: 6 Tables: 2 Supplemental tables: 3 Supplemental figures: 6


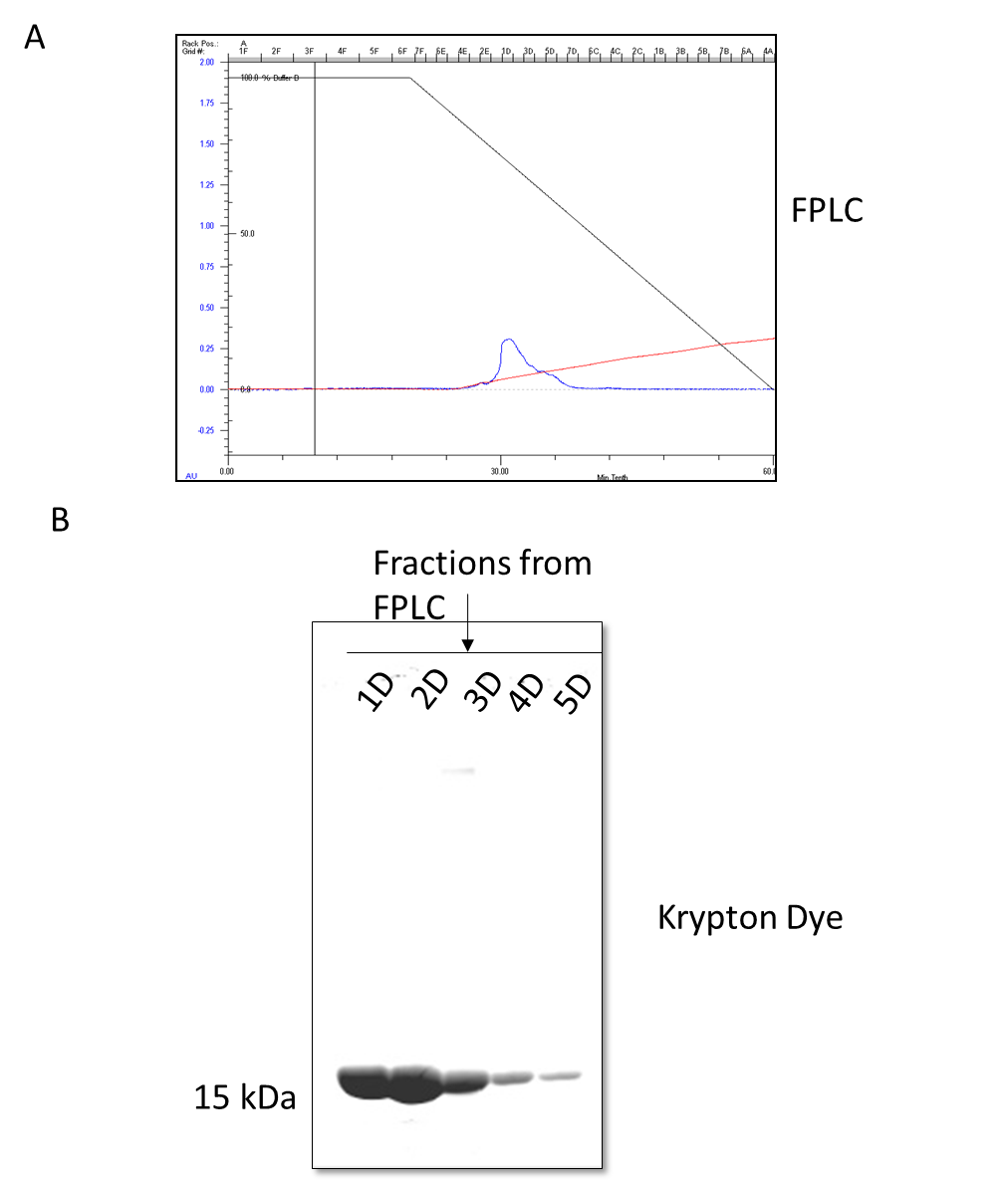


Supplementary Fig. 1. Quality-control of α-synuclein purification. A) FPLC chromatogram showing α-synuclein purity. B) Krypton dye analysis shows different FPLC fractions containing α-synuclein.


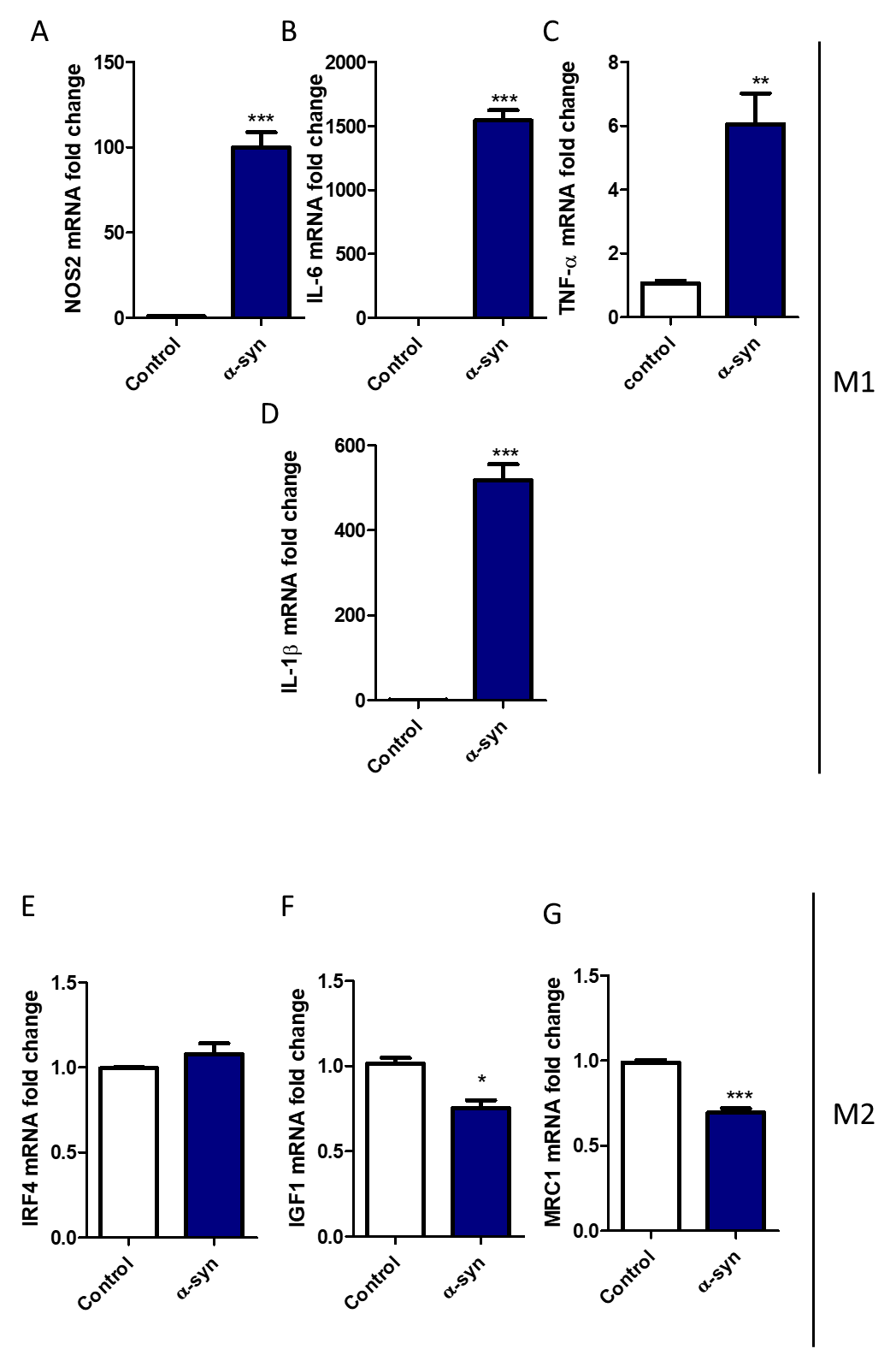


Supplementary Fig. 2. α-synuclein aggregates induce an M1 like phenotype in MMC. A-D) qRT-PCR analysis revealed that α-synuclein aggregates leads to upregulation of M1-like markers A) Nos2, B) IL-6, C) TNF-α and D) IL-1β while downregulating or not altering M2 markers F) IRF4, G) IGF-1, and H) MRC-1. Data analyzed using student’s t test with n=3 for each group. *p<0.05, ** p<0.01, ***<0.005


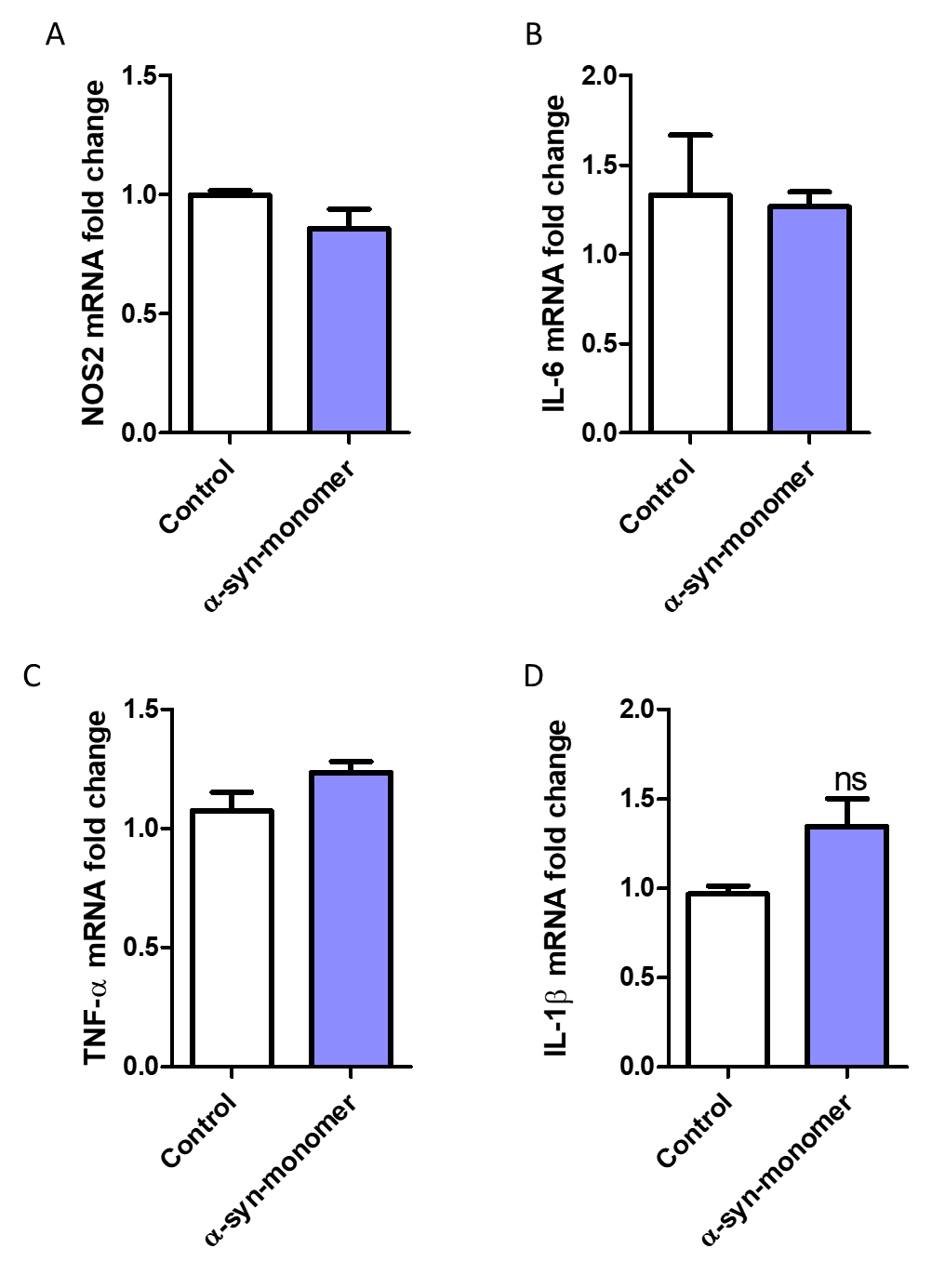


Supplementary Fig. 3. α-synuclein monomer does not induce microglial M-1 like activation. A-D) qRT-PCR analysis revealed that α-synuclein monomers leads to no significant changes of M1-like markers A) Nos2, B) IL-6, C) TNF-α and D) IL-1β in MMC. Data analyzed using student’s t test with n=3 for each group.


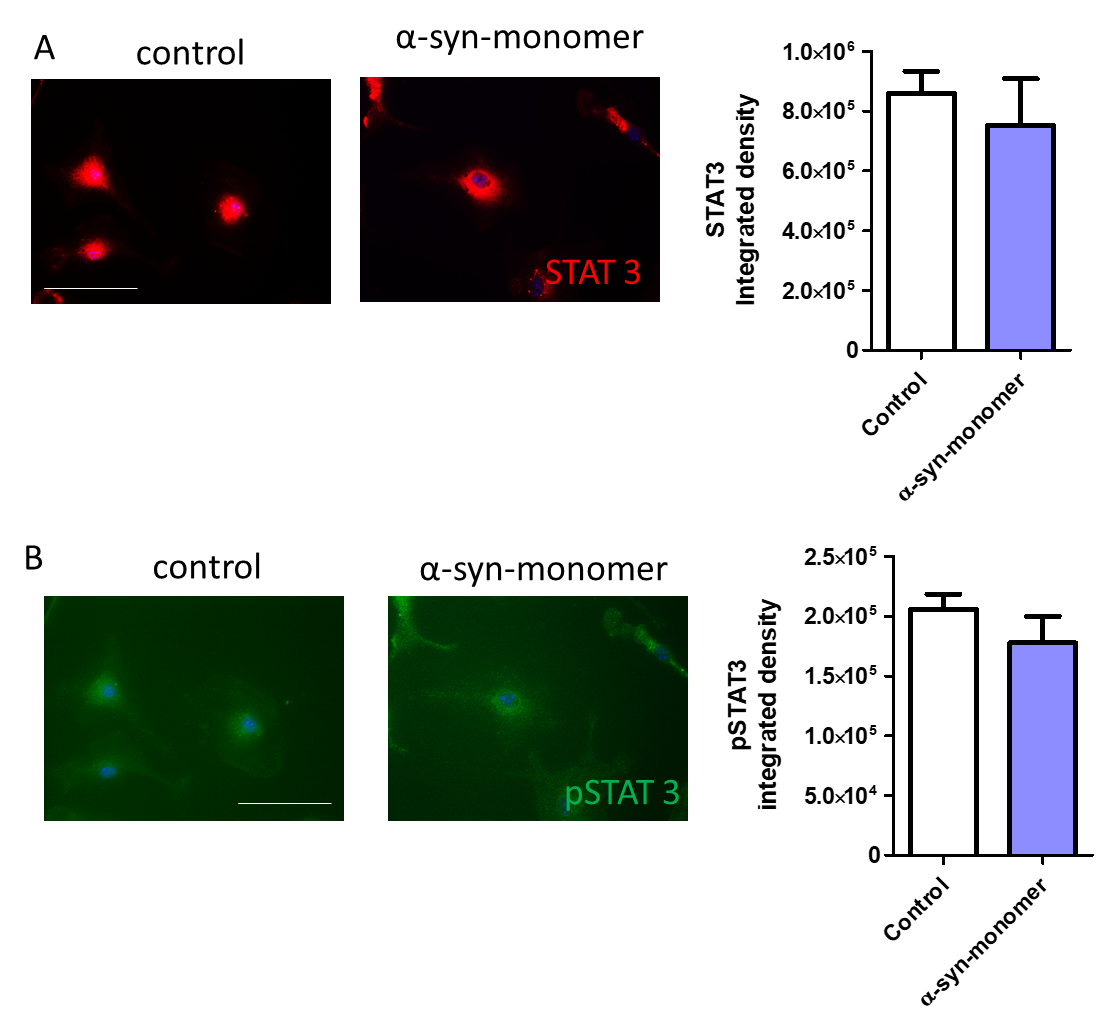


Supplementary Fig. 4. α-synuclein monomer does not induce STAT3 upregulation. A) Immunocytochemical analysis revealed that α-synuclein monomers does not upregulate STAT3 or B) pSTAT3 in primary microglial cells. Scale bar= 20 µM Data analyzed using student’s t test with n=4 for each group.


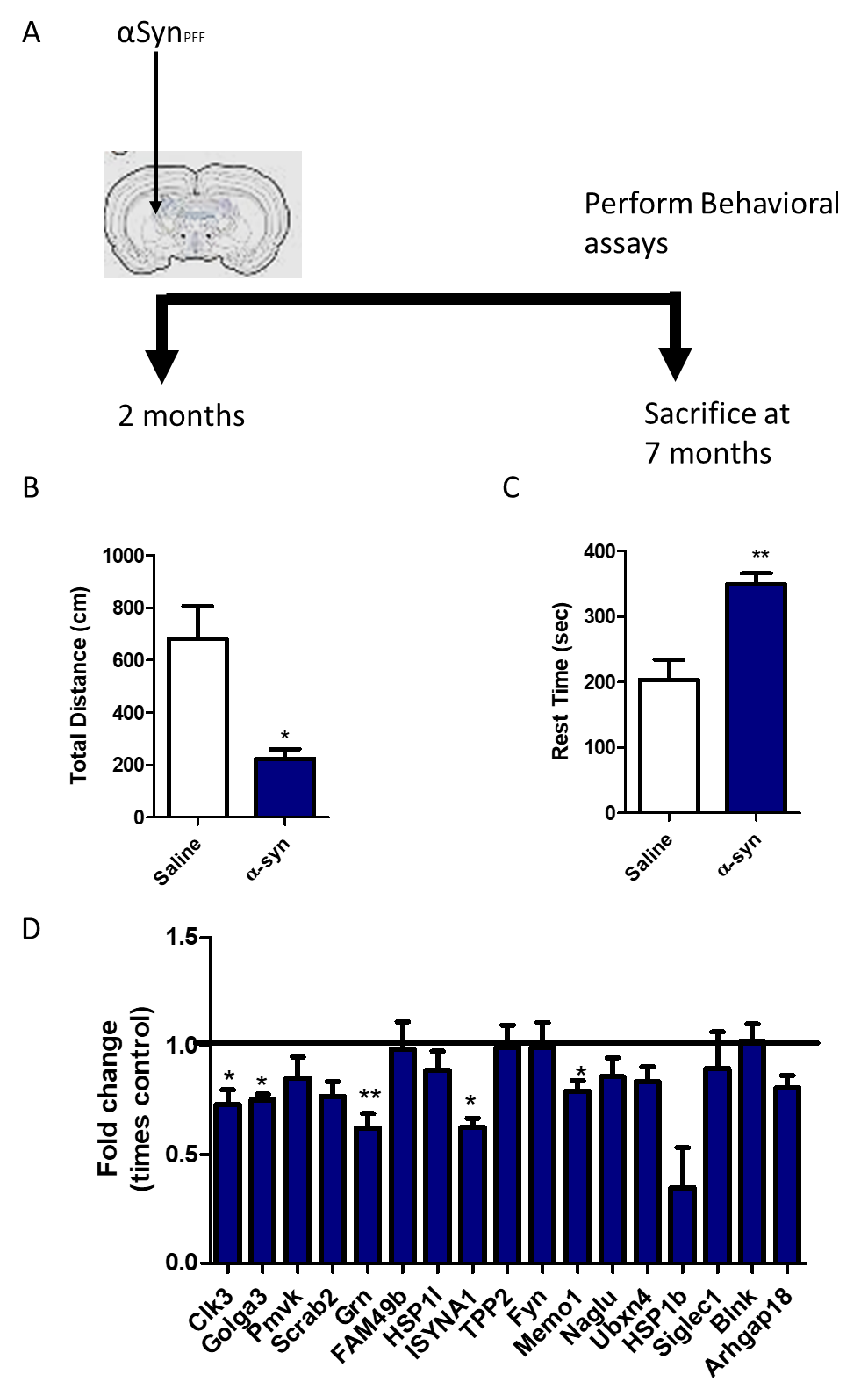


Supplementary Fig. 5. Validation of GWAS hits from proteomics in αSyn_PFF_ model in vivo. A) 8-10 weeks old C57/Bl mice were stereotaxically injected in striatum with α-syn_PFF_ and vehicle and sacrificed 5 months post injection, B-C) Open field versamax test revealed α-synuclein reduced B) total distance covered and B) increased rest time. D) qRT-PCR analysis on SN region of the brain validating some of the hits identified from the proteomic study. Data analyzed using student’s t test with n=3-4 for each group. *p<0.05, ** p<0.01, ***<0.005
